# Nonsurgical Treatment of Peri-Implantitis: Case Series

**DOI:** 10.3390/dj8030078

**Published:** 2020-07-27

**Authors:** Ekaterina Diachkova, Stefano Corbella, Silvio Taschieri, Svetlana Tarasenko

**Affiliations:** 1 Department of Oral Surgery of the Institute of Dentistry, I.M. Sechenov First Moscow State Medical University (Sechenov University), 119048 Moscow, Russia; stefano.corbella@gmail.com (S.C.); silviotaschieri@gmail.com (S.T.); prof_tarasenko@rambler.ru (S.T.); 2 Dipartimento di Scienze Biomediche, Chirurgiche e Odontoiatriche, Università degli Studi di Milano, 20161 Milan, Italy; 3 IRCCS Istituto Ortopedico Galeazzi, 20161 Milan, Italy

**Keywords:** antibiotics, bovine collagen, erythritol powder, nonsurgical treatment, peri-implantitis, radiographic investigation

## Abstract

Peri-implantitis is one of the most important biological complication of dental implants. It has inflammatory nature, proved association with plaque accumulation in peri-implant tissues, and can be progressive on background of several factors, like comorbidity factors and bad habits. The prophylaxis and different methods of treatment were discussed during last 30 years, and surgical and nonsurgical techniques have their foes, benefits, and disadvantages. In this article, we describe the case series of various nonsurgical treatments of peri-implantitis with the use of protocols based on the application of local antibiotics (doxycycline, lincomycin, and erythromycin), mechanical and chemical debridement of dental implant surface, and mini-invasive regenerative technique with injections of bovine collagen. All these three cases demonstrated good results with the maintenance of bone level and absence of clinical signs of inflammation for at least a year according to the X-ray imaging (bone defect volume) and clinic assessments (probing depth, bleeding or suppuration, mucosa color, and pain presence).

## 1. Introduction

Over the last 30 years, dental implants have become the most viable and effective treatment for partial and complete edentulous arches, providing effective masticatory function and esthetics. However, the spread of dental implants in the past decades have brought to light the importance of preventing, diagnosing, and treating peri-implant diseases, because of their important prevalence in the population, over time [1,2,3,4,5].

Under peri-implantitis, now we recognize plaque-associated pathological condition of peri-implant tissues, characterized by inflammatory process of mucosa and progressive bone loss near the dental implants, and can be defined by the identification of several signs such as bleeding or suppuration during careful probing and progressive of peri-implant bone loss beyond the crestal ridge due to initial bone remodeling. In the absence of all these signs, the diagnosis can be provided according the combination of the following criteria: depth of probing not less than 6 mm, appearance of bleeding or suppuration during this measurement test, and the bone loss level with apical direction not less than 3 mm in comparison to coronal intraosseous part of dental implant [6,7,8]. Now, the prevalence of peri-implantitis is higher than it was considered before: during the first 10 years after dental implantation, it varies from 1% to 47% according to a systematic review of Derks and Tomasi (2015) and even about 85% in the work of Dreyer (2018) [9,10], especially in conditions of comorbidity, poor oral hygiene, and bad habits like smoking. 

According to different clinical trials and reviews, the changed vector of World Workshops on the Classification of Periodontal and Peri-implant Diseases, the most effective method of treatment will be several-stages protocol including the resolution of acute inflammation with the help of systemic antibiotics, mechanical and chemical preparation (disinfection) of implant surface in the case of sufficient implant stability and, afterwards, if needed, reconstructive surgery (use of bone grafts, guided bone regeneration, connective tissue transplants, and others) [8]. The nonsurgical treatment, in contrary, is considered as only supporting therapy for cases that are on the margin between loss and the successful treatment [11]. It should be said that nonsurgical treatment could also be adopted to treat patients who refuse surgical treatment for personal reasons and when we need to stabilize soft tissue conditions and bone resorption progression before thinking about surgical treatment. That is why, during the last several years, the scientist and oral surgeons around the world actively discuss the opportunity of post-operative control on the different stages of dental implantation, prophylaxis of peri-implantitis and nonsurgical treatment with the use of different chemical substances (water, saline solution, chlorhexidine) and physical methods (different lasers, ultrasound, ozone, cold plasm, ultraviolet C radiation) for cleaning of dental implant surface, the place of connection “dental implant-abutment,” and pathological bone pockets [12,13,14].

The aim of our article is to present different clinic situations of peri-implantitis and to describe the opportunity of using nonsurgical methods of treatment with satisfying results for dental implant functioning such as supporting measure and the preparation for following surgical treatment. All subjects gave their informed consent for inclusion before they participated in the study. The study was conducted in accordance with the Declaration of Helsinki, and the protocol was approved by the Ethics Committee of Orthopedic Institute Galeazzi (number L2058, 1 June 2019).

## 2. Case Series

### 2.1. Case Report 1

One patient, female, aged 64 years old, presented at the dental clinic of the IRCCS Istituto Ortopedico Galeazzi in Milan, Italy, reporting mild pain and bleeding while performing oral hygiene maneuvers, in the site of the implants placed in mandible more than 10 years before.

The patient was systemically healthy, nonsmokers, and asked for a treatment that may allow her to maintain the implants in site.

We obtained written informed consent from the patient for diagnostics, treatment, and use of photographs.

**Figure 1:**
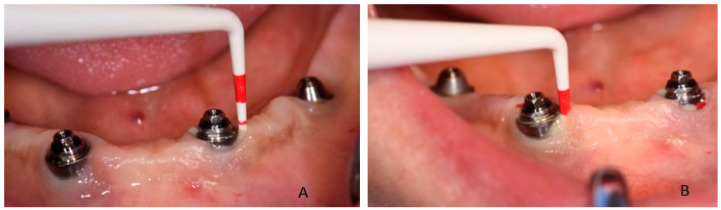
Probing of dental implant area and suppuration in patient during the first visit: (**A**) in 3.2 position and (**B**) in 4.2 position.

**Figure 2:**
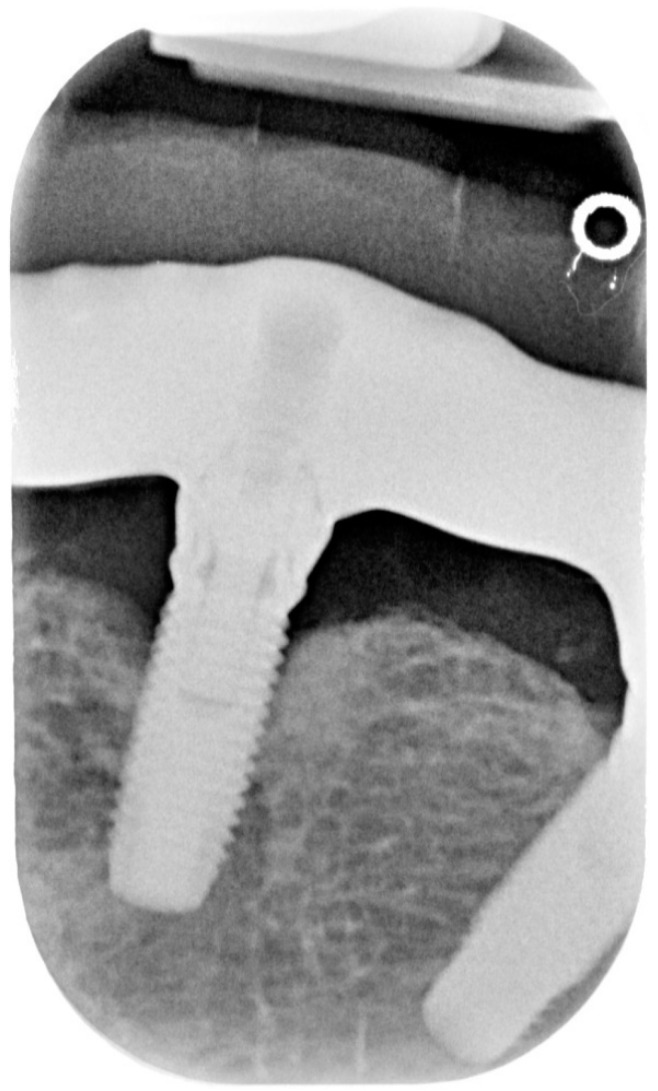
X-ray of dental implant area before treatment.

Clinical examination revealed that the patient was rehabilitated through two full-arch fixed prosthesis, each supported by a combination of two axial and two tilted implants placed in the anterior region of maxilla or mandible, following the All-on-4^®^ protocol [15]. Once the prosthesis was removed, the visual aspect of the peri-implant mucosa surrounding implants placed in 3.2 and 4.2 position revealed the presence of local inflammation (with evident redness and swelling), without any evidence of significant tissue retraction. By the use of one plastic probe (Hawe Perio-Probe™, Kerr Corporation, Orange, CA, USA), we found deep (more than 4 mm) probing depth in all sites of 3.2 and 4.2 implants, with extensive bleeding on probing and suppuration (Figure 1 and Figure 2).

**Figure 3:**
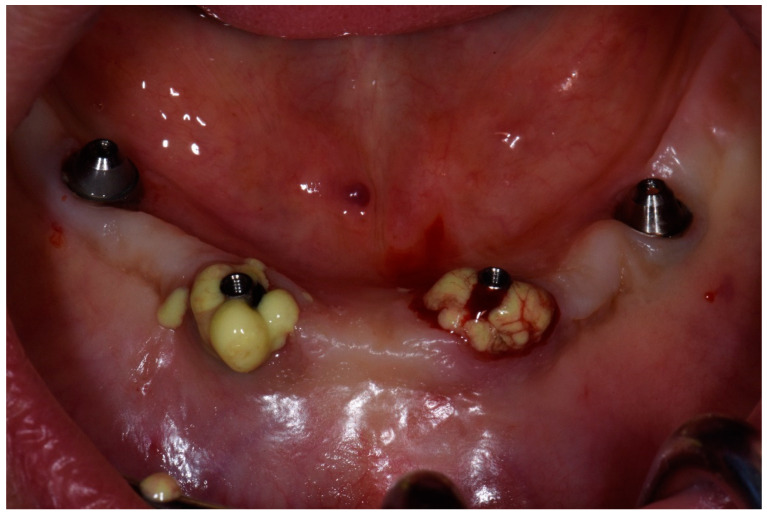
Application of antibiotics with special device around dental implants in positions 3.2 and 4.2.

Before considering performing one surgical procedure in order to treat the pathology (by regenerative or resective surgery), the authors decided to perform nonsurgical treatment, following the patient’s will (who rejected the surgical treatment for the first instance) and to evaluate the results over time, with particular attention to the soft tissue healing following the treatment. We proposed the use of topical applications of 14% doxycycline (Ligosan^®^, Kulzer, Hanau, Germany). We performed two professional treatments in two visits within 1 week. After local anesthesia with articaine 4% and epinephrine 1:200,000, the antibiotic was placed directly into the pocket with a specific device. The injection was given gently, with a specific and dedicated sterilized device, until we clearly visualized the antibiotic spilling outside of the pocket after filling it completely (Figure 3). After cleaning the surface of the abutments from the residues of the antibiotic, the prosthesis was screwed again. The patient was instructed to avoid any trauma involving the peri-implant mucosa and to rinse with 15 mL 0.2% chlorhexidine twice a day for 7 days after each treatment.

**Figure 4:**
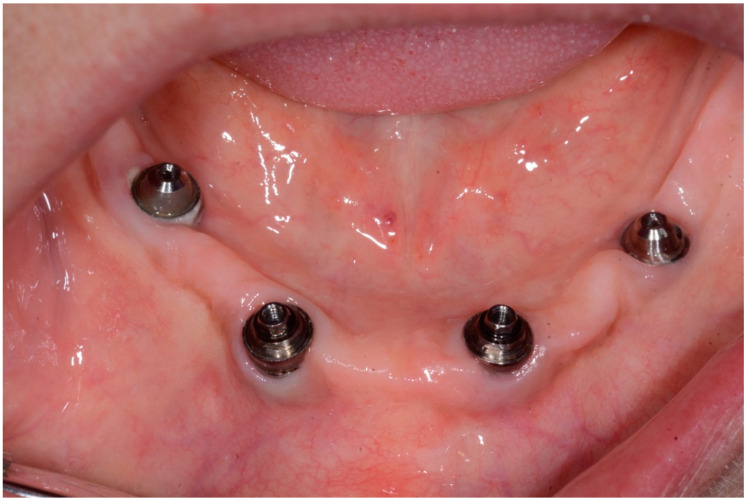
Condition of soft tissues around dental implants in positions 3.2 and 4.2 in 6 months after treatment.

**Figure 5:**
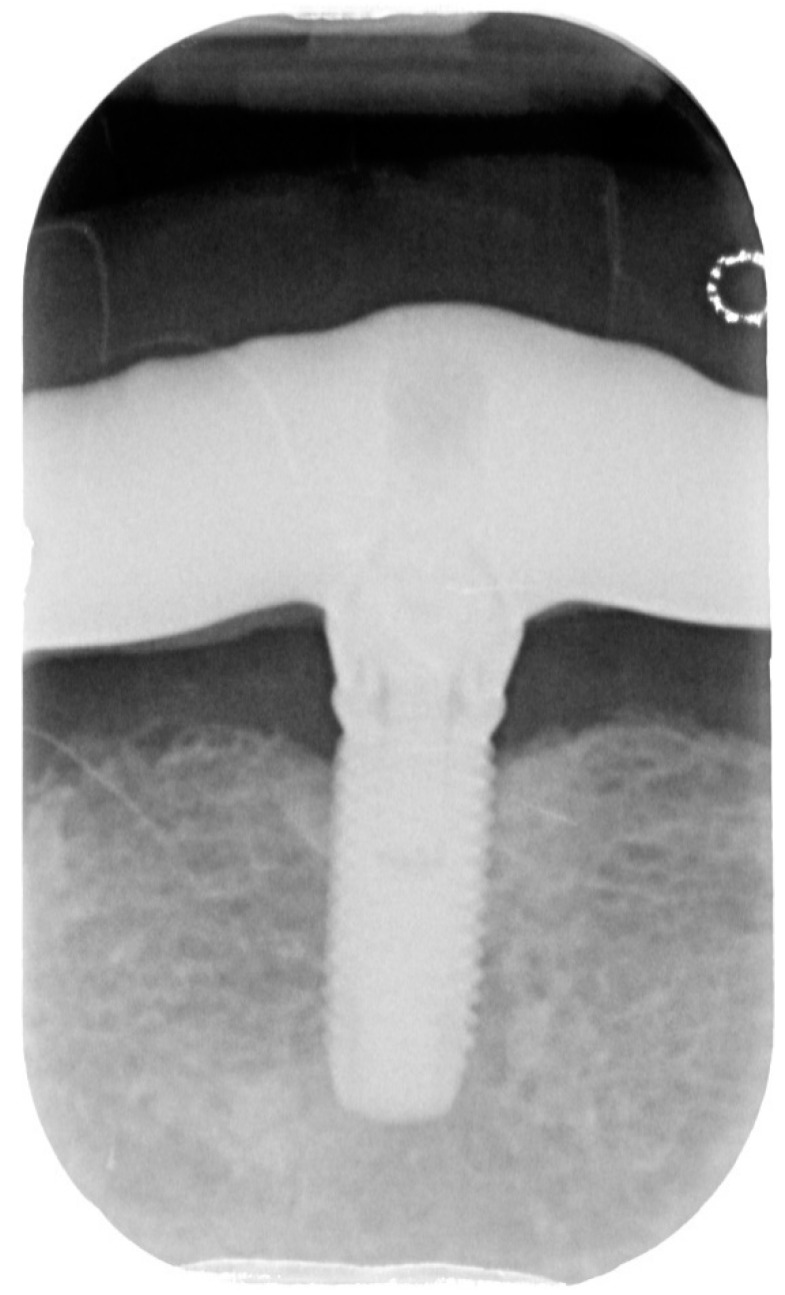
X-ray of dental implant area after treatment.

The postoperative period was completely uneventful, and the patient was followed-up after 1 month, after 3 months, and after 6 months to evaluate the evidence of further bone resorption and the absence of signs and symptoms of inflammation (bleeding on probing, suppuration, swelling, and redness). The probing index after the treatment decreased of 2.1 mm (on average). After 6 months, the clinical situation of the soft tissue was stable (Figure 4), without signs of inflammation and we appreciated no further bone resorption (Figure 5). The patient would be followed up every 3 months to evaluate eventual further surgical treatment.

### 2.2. Case Report 2

One patient, male, aged 23 years old, presented at the dental clinic of the Department of Oral Surgery of the Institute of Dentistry of Sechenov University, Moscow, Russia, reporting mild pain, unpleasant smell from oral cavity, and suppuration while performing oral hygiene maneuvers, in the site of the implant placed in maxilla more than 5 years ago.

The patient was almost systemically healthy (class II according American Association of Anesthesiologists because of the presence of heart disease in the stage of the investigation (failure of the heart rhythm), the patient was severe smoker (>20 cigarettes per a day), and asked for a treatment that may allow him to maintain the implants in site.

We obtained written informed consent from the patient for diagnostics, treatment, and use of photographs.

**Figure 6:**
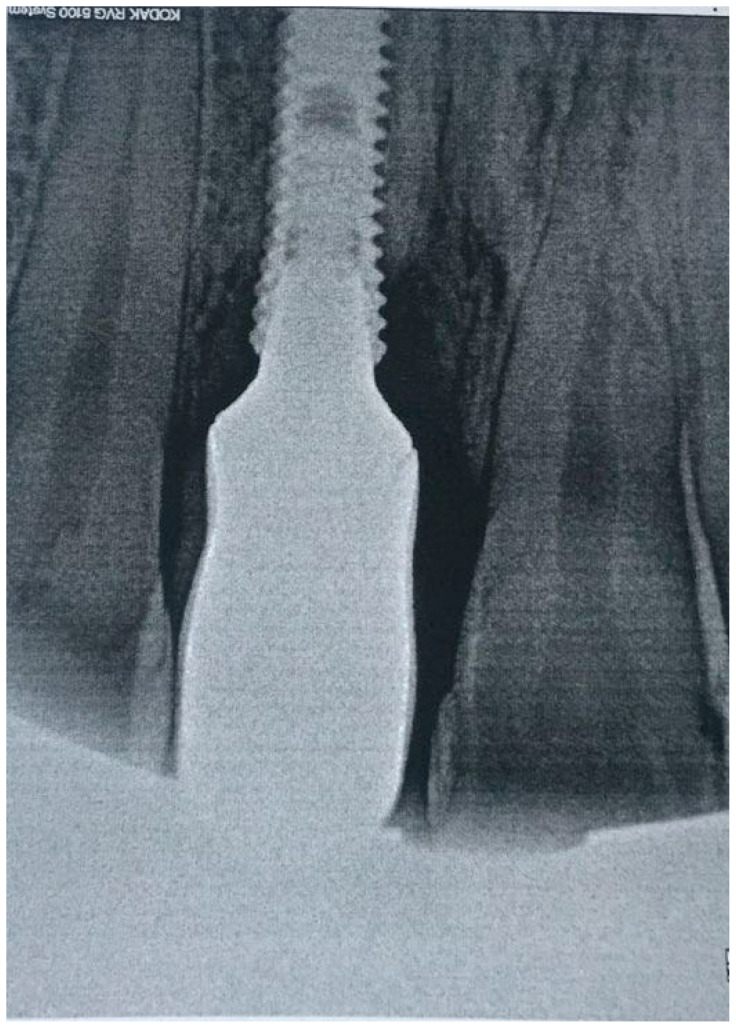
X-ray of patient before treatment in position 1.1 of dental implant.

Clinical examination revealed that the patient was rehabilitated through one crown with gum mask cemented on one dental implant placed in 1.1 position and had the presence of local inflammation (with evident redness and swelling) with significant soft tissue retraction. By the use of one plastic probe (Hawe Perio-Probe™, Kerr Corporation, Orange, CA, USA), we found deep (more than 4 mm) probing depth in all sites of 1.1 position of dental implant improving on radiological investigation, with intensive bleeding on probing and suppuration (Figure 6).

**Figure 7:**
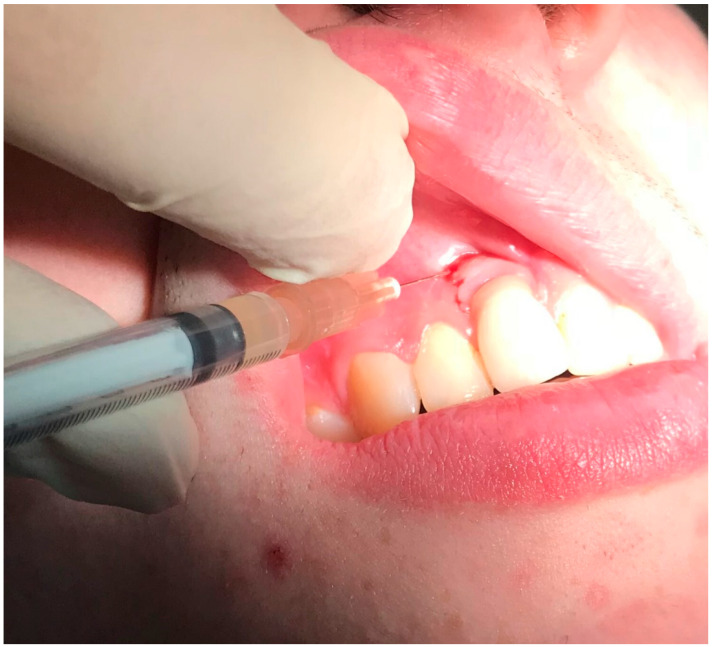
Application of 7% bovine collagen gel in patient in position 1.1 of dental implant.

Respecting patient’s will, and after a thorough discussion of pros and limits of the treatment options, we decided to perform nonsurgical treatment and monitoring patient over time with the special attention to stabilize the condition of soft and bone tissues. After manipulation, we proposed the use of topical applications of 30% lincomycin hydrochloride (Lincomycin^®^, DalChemPharm, Khabarovsk, Russia) on the first day of treatment after professional oral hygiene maneuvers (due to the stability of dental implant, ultrasonic cleaning of plaques was performed with Airflow powder and handpiece (Airflow Prophylaxis Master, EMS, EMS, Switzerland) in the area of all teeth and dental implant, using the PI EMS headpiece for Piezon with the plastic cover). We performed two treatments with the use of 7% bovine collagen injections (Collost^®^, BioPHARMAHOLDING, Moscow, Russia) in two visits within 2 weeks. The choice of treatment was based on the good performances demonstrated by collagen on peri-implantitis and on bone healing in experimental research [16,17]. After local anesthesia with articaine 4% and epinephrine 1:200,000, the antibiotic was placed directly into the pockets near the dental implant with a syringe with cannula 26G followed by injections of 7% bovine collagen inside bone pockets and gum with syringe and cannula 29G. The injection was given gently, until we clearly visualized the gel spilling outside of the pocket after filling it completely (Figure 7). 

After cleaning the area of procedure, we did gum binding with ointment (Solcoseryl^®^ dental adhesive paste, MEDA Pharma, Moscow, Russia). The patient was instructed to avoid any trauma and overheating involving the peri-implant mucosa, to rinse with 15 mL 0.2% chlorhexidine 3 times per a day for 7 days after each visit, and to maintain the satisfactory level of oral hygiene. The crown was not removed and changed for new one according to the will of patient, the absence of opportunity from his side to pay for prosthetic treatment. Also, we considered this case like indication for radical treatment but were limited with specific factors.

**Figure 8:**
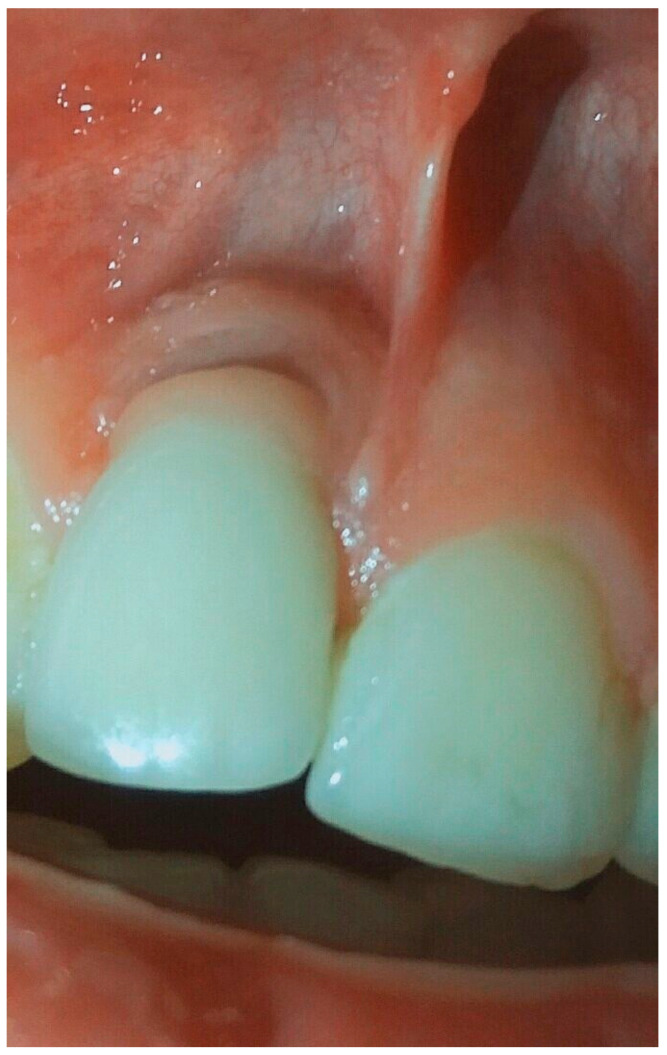
Condition of soft tissue near dental implant in 1.1 position after treatment in 6 months.

**Figure 9:**
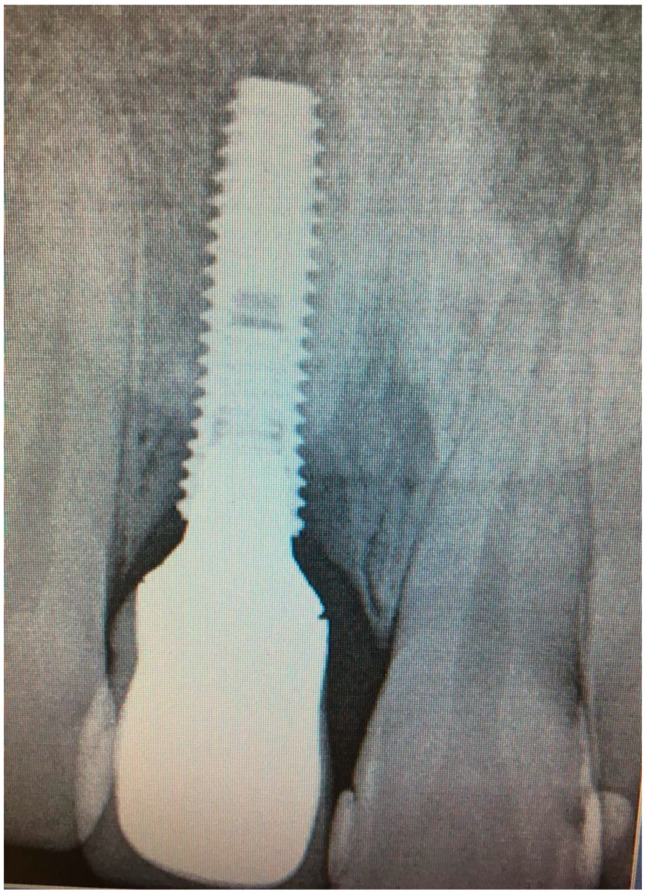
X-ray of patient in 6 months after treatment.

The period after treatment was completely light, and the patient was followed-up after 1 month, after 3 months, and after 6 months to control the condition of soft and bone tissues according the presence of any signs of disease progression. After 6 months, the clinical situation of the soft tissue was stable, without signs of inflammation and we appreciated no further bone resorption (Figure 8 and Figure 9). The patient would be followed up every 3 months to evaluate the condition of peri-implant tissues and to prepare for future surgical treatment.

### 2.3. Case Report 3

A man, aged 68, nonsmoker, without any uncontrolled systemic disease (ASA-2 following the classification of the American Society of Anesthesiologists that may increase the risk of peri-implant or periodontal diseases, i.e., diabetes or immunological impairment) was presented to the attention of the University Dental Clinical Department, IRCCS Istituto Ortopedico Galeazzi with the symptoms of peri-implantitis and was referred to the implant-supported rehabilitation. 

The patient showed a prosthetic rehabilitation on four implants (All-on-4^®^) of the lower and upper jaw. Implants were placed 8 years before, in the same Dental Clinical Department.

We obtained written informed consent from the patient for diagnostics, treatment, and use of photographs. 

**Figure 10:**
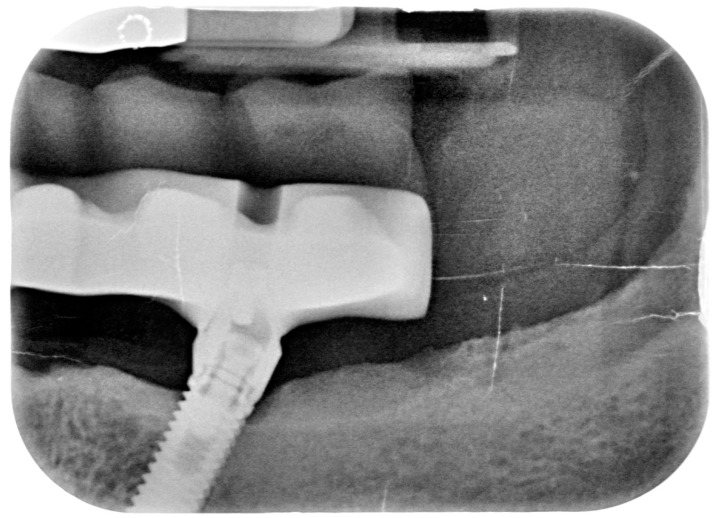
X-ray of 3.5 dental implant in patient before treatment.

**Figure 11:**
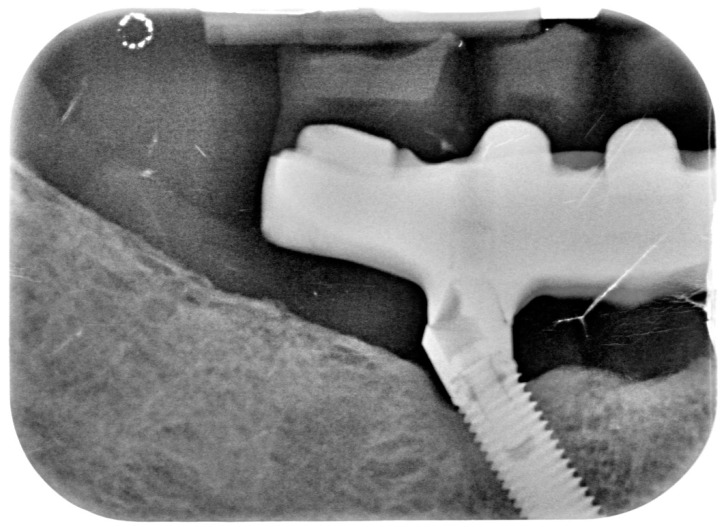
X-ray of 4.5 dental implant in patient before treatment.

After removing the prosthetic rehabilitations, diagnosis of the soft tissue was done. Peri-implant assessment (PPD, TL, BI, PI, and mobility) and, subsequently, assessment of color, contour, consistency of soft tissues was performed. Individual oral hygiene instructions including re-education and motivation if needed was done. Bidimensional radiographic assessment (with periapical and panoramic radiograph) was performed (Figure 10 and Figure 11).

The clinical assessment allowed finding a probing depth of 6 mm mesial/circumferentially to 3.5 with bleeding in the two lower jaw distal implants and a probing depth of 4–2.5 mm mesial/circumferentially with bleeding in the two lower jaw central implants. In the upper jaw, a probing depth of 1–2 mm was found. None of the implants showed mobility.

A bidimensional radiographic assessment of the lower jaw and upper jaw implants (with periapical and panoramic radiograph) was done. In the lower jaw implants, a concave bone resorption can be observed of about 4 mm mesial and distal to both distal implants (3.5 and 4.5). No bone resorption was revealed on the two lower jaw central implants and on the four implants positioned in the upper jaw.

A diagnosis of peri-implantitis of the two lower jaw distal implants and of mucositis in the two lower jaw central implants was reported. 

The patient was informed about his clinical conditions.

Treatments alternative (surgical and nonsurgical) were proposed, which would have implied the use of different antibacterial products and specific instruments/device [18,19].

The subject was informed about advantages and disadvantages of the proposed alternative.

Upper jaw implant prosthetic rehabilitation received supragingival biofilm removal using erythritol powder (AF Plus Powder, Airflow Handpiece, and Airflow Prophylaxis Master device from EMS, Nyon, Switzerland). The device was used as per recommendations: power set at 30–60% and water set at maximum. Lower jaw implants were treated depending on the pathology diagnosed.

Supra and subgingival calculus/plaque around implants was removed using PI tip (EMS, Nyon, Switzerland) mounted on a Piezo LED Handpiece (Airflow Prophylaxis Master, EMS, Nyon, Switzerland) set at 40% power and maximum water. Subsequently, biofilm removal was achieved using erythritol powder (AF Plus Powder, Perioflow Handpiece mounting a specific nozzle, EMS, Switzerland). Additionally, in this case, the device is to be used as per recommendations: power set at 30–60% and water set at maximum. The Perioflow Handpiece should be used in a vertical direction with intermittent repetitive movements towards the occlusal or incisal surface. The instrumentation time at each aspect (i.e., mesial, distal, vestibular, and oral) was limited to 5 s per site (four to six sites per implant), as recommended by the manufacturer [20].

Individual oral hygiene instructions were given specifically to manage plaque accumulation at implant sites using a soft toothbrush, interproximal brushes, toothpicks, or dental floss at each time point when subjects were examined. Similarly, consistent with good clinical dental practice, oral hygiene instructions were given to enhance oral hygiene in natural teeth. This was also performed, if needed, at each follow-up visit [21,22,23].

The follow-up was performed at 1 month, 3 months, and 1 year.

Clinical assessment of PPD, TL, BI, PI, and mobility was collected at each follow-up visit. Periapical radiograph was taken at 6 months (±14 days) and 12 months (±14 days).

**Figure 12:**
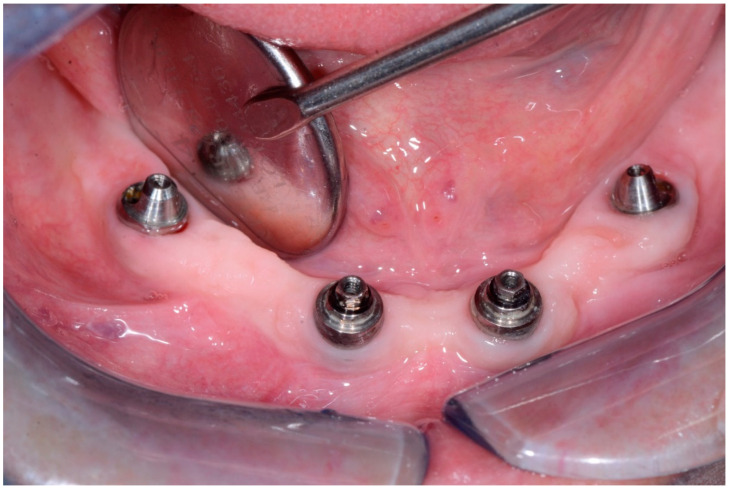
Condition of peri-implant tissues in patient in 6 months after treatment.

In 1 year the clinical assessment allowed to find a probing depth of 1.5–3 mm mesial/circumferentially without bleeding in the two lower jaw distal implants and a probing depth of 0.5–1 mm mesial/circumferentially without bleeding in the two lower jaw central implants (Figure 12). 

**Figure 13:**
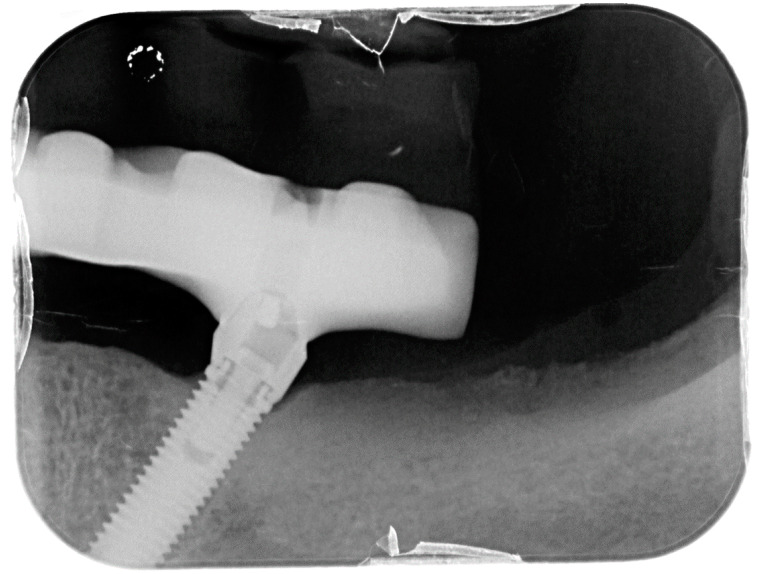
X-ray of 3.5 dental implant in patient in 6 months after treatment.

**Figure 14:**
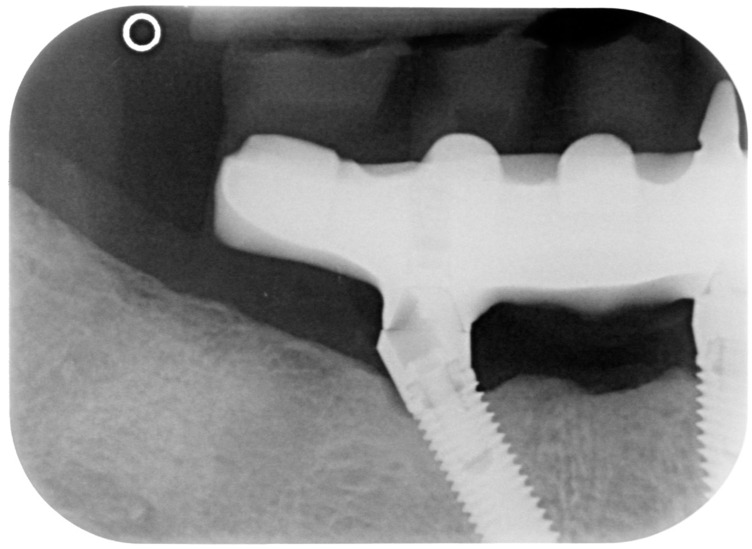
X-ray of 4.5 dental implant in patient in 6 months after treatment.

Bidimensional radiographic assessment of the lower jaw implants (with periapical and panoramic radiograph) was done. In the lower jaw, a concave bone resorption of about 2.5 mm mesial and distal to both distal implants were observed, and there was no bone resorption in two central implants (Figure 13 and Figure 14).

## 3. Discussion

In presented cases, the authors attempted to demonstrate different nonsurgical methods of peri-implantitis that can be easy repeated and be available almost in all clinics. During the treatment, there should be constant communication between doctor and the patient, and it is fundamental to obtain full compliance and full cooperation of the patients. 

Dental implant placement and prosthetic loading lead to remodeling of bone tissue and some extent of “physiological” bone resorption. According to several experimental studies, this process may depend on the presence of microgap in system dental implant-abutment and its location. There is opinion that decrease of bone level in healthy tissues can relate to the dental implant design. Thus, the same authors tell that majority of dental implants (approximately 75%) can support around enough bone tissue due to active osteointegration process. The bone loss of more than 1 mm associates with hidden mucosa inflammation even in condition of the absence of clinical signs of mucositis [24]. 

Poor oral hygiene can lead to formation of bacterial plaque near the dental implants that appears the potential predictor for development of mucositis and peri-implantitis [8]. The correlation between biofilm accumulation near dental implants in experiment and appearance of inflammatory process (mucositis exactly) was demonstrated in humans. Indeed, even after a long uneventful period of peri-implant tissues health, in experimental settings, it was demonstrated that just 3 weeks (or even less) without oral hygiene are sufficient to cause peri-implant tissue inflammation with biofilm accumulation and bleeding on probing [25]. The prevalence of mucositis in people that abolished personal oral maneuvers was found to be up to 48% [26]. We should highlight that measurements of periodontal and “hygiene” indexes may allow to adequately control the area of operation and timely prevent or treat early signs of peri-implant tissue inflammation.

Due to the same reason, the mechanical control of microbial biofilm near the dental implants now is considered as of paramount importance for managing the peri-implant tissue diseases, from both sides, i.e., professional dentist as well as patients themselves [19]. 

This point of view is useful in cases when patients are not ready to resective or other “radical” surgical treatments while the presence of clinical signs of peri-implantitis require some procedures to prevent the loss of dental implants and the spreading inflammatory process. As minimal procedures, that can be provided, are presented in our work (the cases 1 and 3 and some part of case 2) and include the application of antibiotics with local high activity for control of microbial biofilm and prevention of further spreading of infection. The benefit of local applications of antibiotics was mentioned in work of Wang (2020) [12], although the systemic use of antibiotics for the similar situation was also discussed [19]. 

Hence, already in 2010, Lang has pointed on the fact that only mechanical removal of microbial biofilm is not sufficient procedure for good results in case of presence of inflammation in peri-implant tissues and has limitations in improvement of the condition. The same situation can be shown even during additional cleaning of surface of dental implants, which can lead only to a little decrease in bone loss level but not its end. According the results of some case reports, published in literature almost 30 years ago, the authors have demonstrated the clinical advantages of pathological pockets irrigation with antiseptics and antibiotics after mechanical cleaning of dental implant area for 12 months [27]. The best results were proved in one clinical trial describing nonsurgical treatment of peri-implantitis (that included photodynamic therapy besides oral hygiene maintenance and mechanical debridement) [19]. However, the authors did not find strong correlation between conservative methods and clinical and microbiological improvement [19]. According to some research, the decontamination of dental implant surface with the different kinds of laser can stop the spreading of inflammatory process and in some cases, even arrest it [12,13,19]. 

Using only decontamination methods for treatment of peri-implantitis failed to show good results according to clinical, radiological, and microbiological criteria due to only temporary recovery but they are rather effective in combination with surgical methods. Indeed, surgical treatment remains the main effective option in treatment of peri-implantitis, thus allowing to prevent further bone loss. The nonaugmentative techniques (resective surgery) direct to decrease or removal of pathological bone pockets around dental implants, which change the bone contour and soft tissue architecture with repositioning flaps with or without dental implant surface remodeling. Implantoplasty allows to provide decrease in clinical symptoms of peri-implantitis such as bleeding during probing and depth of bone pockets that can be proved with X-ray imaging. In the cases of exacerbation of process after nonsurgical treatment and presence of permanent bone defects around dental implant, there is need to perform the regenerative techniques, which demonstrated to lead to stable results over time. Although the variance of using materials (autogenous bone, xenograft, etc.) is wide, there is no priority in long-term clinical advantage for any. It is considered that the best treatment algorithm must include keratinized gum restore, bone level support, and plaque control that can be provided with use of apical reposition flap in combination with free gingival transplant [18].

In a systematic review, Suárez-López Del Amo F. et al. (2016) analyzed different proposed approaches for peri-implantitis and mucositis treatment and found no limitations for them but effectiveness. Thus, outcomes of nonsurgical treatment with laser and other technique approximately are equal for decreasing the inflammation, however, for regeneration of the bone, they are less effective than regenerative techniques because according to the literature, they allow to restore about 1 mm on bone in the area of peri-implant defects [28]. Decision about method of treatment lays on surgeon but some recommendations now are given with World Workshop for last 3 years on FDI Congress [8,9,18].

Due to the will of patient and limits of treatment opportunities, we decided that using regenerative component, as it was described in our case 2 during nonsurgical treatment of peri-implantitis, can be useful in some cases for maintaining of peri-implant tissues volume.

## 4. Conclusions

Obviously, the present study will not allow the authors to provide general guidelines about the nonsurgical treatment of peri-implant disease neither to generalize the results obtained in the presented case report to the general population. More subjects are needed to provide support to the obtained results. Moreover, we should highlight that in all the cases, the decision of performing nonsurgical treatment alone was made on the basis of patients will, since, in general terms, peri-implantitis often requires a surgical approach to be effectively treated, on the basis of the existing literature.
